# Supplementary material for: Reprocessable Polybenzoxazine Thermosets with High Tgs and Mechanical Strength Retentions Using Boronic Ester Bonds as Crosslinkages
Source: Polymers (Basel). 2022 May 31;14(11):2234. doi: 10.3390/polym14112234 (PMC9182748; doi:10.3390/polym14112234)
Supplement: Supplementary file 1 [file polymers-14-02234-s001.zip › polymers-1739274-supplementary.pdf]

Supporting Information

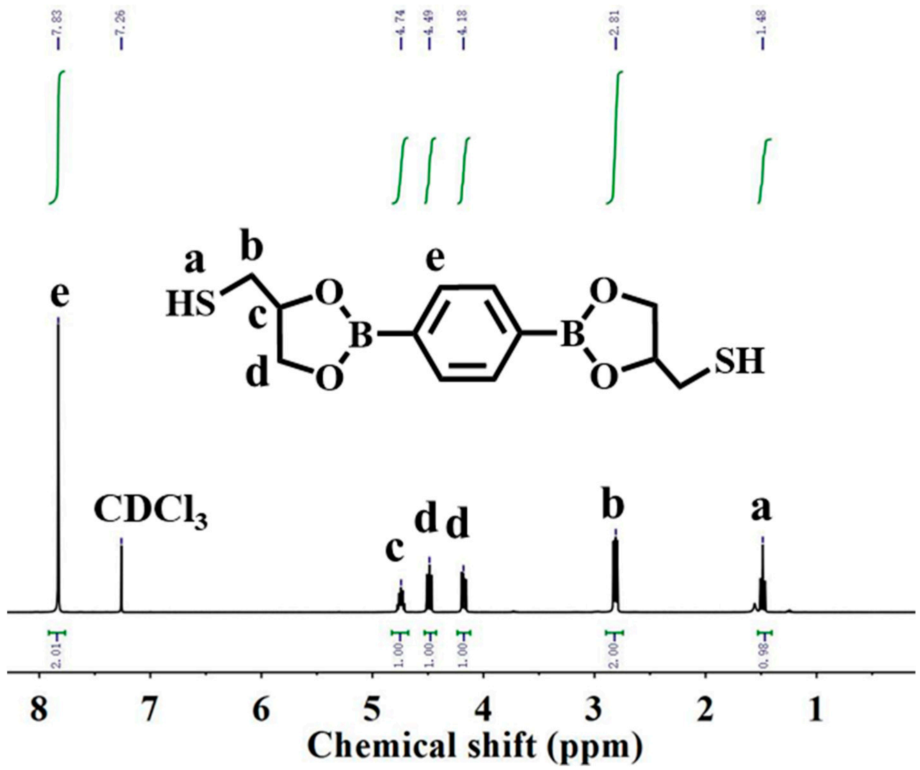

Figure S1. ^{1}H NMR spectrum of BDB.

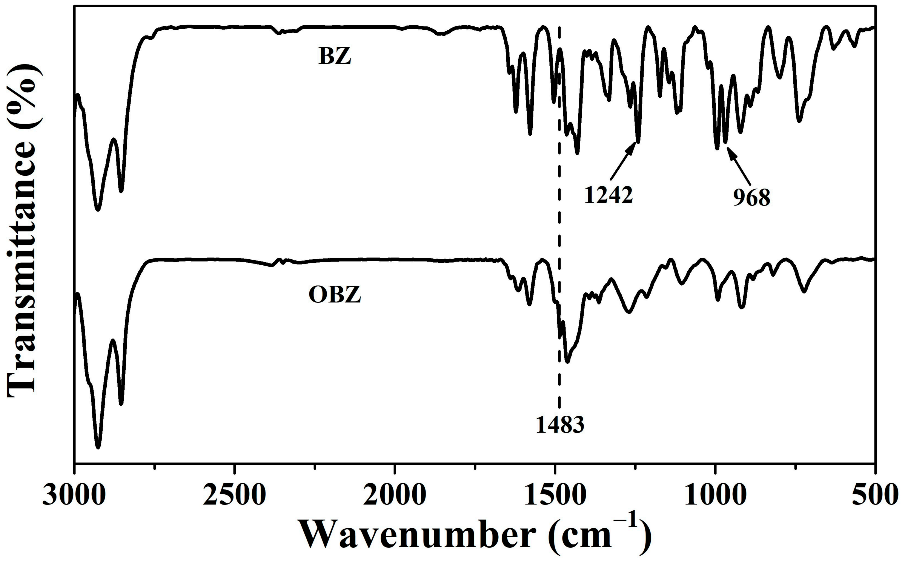

Figure S2. FTIR spectra of BZ and OBZ.

Table S1. Tensile strengths and elongations at break of original and reprocessed OBZ-BDB samples.

Sample	Tensile strength (MPa)	Elongation at break (%)
original	31.0±1.3	8.4±0.5
1st	30.6±0.9	8.1±0.2
2nd	29.6±1.1	7.1±0.3
3rd	26.0±1.5	6.4±0.7
